# Apoptosis triggers the release of microRNA miR-294 in spent culture media of blastocysts

**DOI:** 10.1007/s10815-020-01796-5

**Published:** 2020-05-21

**Authors:** Dimitra Makri, Panagiota Efstathiou, Eftychia Michailidou, Walid E Maalouf

**Affiliations:** School of Medicine, Division of Child Health, Obstetrics, and Gynaecology, Queen’s Medical Centre, University of Nottingham, Nottingham, UK

**Keywords:** microRNAs, miRNAs, Embryo, Spent culture medium, Apoptosis, Time-lapse, Noninvasive

## Abstract

**Purpose:**

To study whether members of the miR-290-295 cluster in spent culture medium (SCM) of embryos are correlated with morphokinetics and apoptosis.

**Methods:**

Cryopreserved 1-cell stage mouse embryos were cultured to the blastocyst stage, development was monitored by time-lapse, 59 SCM were collected, and miR-291a and miR-294 were detected with polymerase chain reaction (PCR). Blastocysts were immuno-stained for sexing (H2AK119ub) and for apoptosis (TUNEL). Each embryo and SCM were individually processed. Correlations were run between the miRNAs and developmental events (t2, t3, t4, t5, t8, tSB, tB, ECC2, ECC3, s2, s3, dB) and apoptosis (apoptotic cells/total cell number %). MiR-294 SCM and cell levels were compared in 40 blastocysts. Apoptosis was induced in 15 blastocysts with UV radiation and SCM samples were analyzed for miR-294.

**Results:**

MiR-291a and miR-294 are released in variable levels by mouse blastocysts. Their release is similar between male and female embryos. No significant correlations were found between these miRNAs and development. MiR-294 was significantly positively correlated with apoptosis (*r* = 0.560, *p* < 0.001). Cellular expression was lower in blastocysts that released miR-294 in high levels compared with null, low, and medium release embryos (*p* < 0.01). UV radiation caused apoptosis which triggered higher secretion of miR-294 in 15 blastocysts versus 13 control embryos (*p* < 0.01).

**Conclusion(s):**

MicroRNAs are important regulators of preimplantation development. Apoptosis triggers the release of miR-294 by blastocysts which possibly serves a secretory role for embryo-maternal communication. SCM miRNA analysis is possible for individually cultured embryos and future studies can investigate miRNAs as noninvasive markers of embryo quality.

**Electronic supplementary material:**

The online version of this article (10.1007/s10815-020-01796-5) contains supplementary material, which is available to authorized users.

## Introduction

MicroRNAs (miRNAs) are small (~ 22 nucleotides), single-stranded, non-coding, and highly conserved RNAs that regulate important cell processes such as stress signaling, cell cycle progression, development, differentiation, and apoptosis [1]. During the differentiation of mammalian gametes and the subsequent embryo development, miRNAs are dynamically expressed in a tightly controlled and time-dependent fashion [2–5]. These molecules, in synergy with other factors, regulate and control normal growth and development of the preimplantation embryo. In the last decade, the roles of miRNAs in regulation of mammalian reproduction are under research. Initially McCallie et al. [6] reported differences in embryo miRNA expression relating to parental fertility status. Significant under-expression of six miRNAs, all involved in cell growth, proliferation, and differentiation, was observed in blastocysts of infertile patients compared with fertile [6]. Moreover, aneuploid embryos express aberrant levels of miRNAs compared with euploid ones meaning that, possibly, miRNAs can serve as markers of embryo quality [7].

Interestingly, miRNAs are not only present intracellularly but also released via exosomes and apoptotic bodies and bound to lipids or proteins [8] and cell-free miRNAs are present in body fluids such as serum and amniotic fluid [9]. Mammalian embryos also release miRNAs in the surrounding micro-environment [10], and over the last few years, analysis of spent culture media (SCM) for the presence of miRNAs has emerged as a noninvasive method for assessing embryo quality. For instance, in bovine, miRNAs -10b, -44, -45, -24, 191, and 148a are present in higher levels in SCM of degenerate embryos compared with blastocysts [11, 12]. Additionally, miR-30c and miR-10b possibly act on cell cycle regulation since they are abundant in SCM of slow-cleaving embryos [12]**.** Furthermore, miR-191 in SCM is associated with aneuploidy and miR-191, miR-372, and miR-645 are highly released by embryos that fail to lead to a live birth in humans [13]. It is also known that miRNA release via exosomes is an active process which promotes the embryo-maternal communication at the peri-implantation window [14, 15]. In bovine, embryo-secreted miRNAs are uptaken by maternal cells and cause a transcriptomic response meaning that they are involved, and perhaps necessary, to prepare the endometrium for embryo implantation [16]. Indeed, this has been shown in humans where miR-661 is taken up by endometrial cells causing downregulation of adhesion and invasion factors (*PVRL1*, *MTA2*) which in turn affects embryo implantation [17]. Additionally, miR-20a and miR-30c are highly secreted by euploid implanted embryos compared with non-implanted [18]. These studies not only show that miRNAs regulate embryo-uterine interaction but also suggest that miRNAs are valuable noninvasive biomarkers for prediction of clinical outcomes.

Despite the encouraging evidence, the exact mechanisms via which miRNAs regulate embryo development are poorly understood. Currently there is limited evidence regarding the involvement of miRNAs on key processes controlling preimplantation development. Considering the lack of information, we aim to provide answers on the roles of specific miRNAs belonging to the miR-290-295 cluster in two processes that are essential for normal embryonic growth: developmental dynamics/kinetics and apoptosis. More specifically, we examine whether certain developmental events occurring at the preimplantation stages are linked to the active release of miR-291a and miR-294. Additionally, we investigate whether blastocysts with impaired DNA quality release these miRNAs in differential levels than good-quality embryos. Our aim is to provide a mechanistic explanation on the fact that embryos release certain miRNA in varying levels, which can be detected in SCM and perhaps reflect on further developmental potential. Overall, we hypothesize that miR-291a and miR-294 are released in different levels by blastocysts and their expression is associated with differences in morphokinetics and apoptosis.

## Materials and methods

### Ethics

This study was exempt from ethical approval, as frozen embryos were purchased and cultured up to the blastocyst stage according to the Animals Scientific Procedures Act 1986 (ASPA) for the protection of animals.

### Study design

For the purposes of this study, it was essential to have a detailed picture on the association of the miRNAs with development and apoptosis. Therefore, we analyzed individual samples of SCM deriving from singly cultured embryos, a technical approach which has not been reported previously. MiRNA data were then associated with morphokinetic information and apoptotic percentage for each embryo individually. Morphokinetics were assessed using time-lapse incubation technology because it allows to continuously monitor the embryos and annotate dynamic events, cleavage patterns, and timings of events and extract detailed descriptive information about the development of each embryo individually. Embryo quality was assessed by using apoptosis detection staining and calculating the percentage of apoptotic cells at the blastocyst stage.

### Mouse embryo thawing and time-lapse culture

**Fig. 1:**
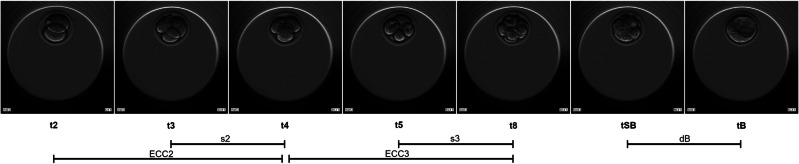
Time-lapse snapshots of mouse embryo development. The exact timings of 2-cell, 3-cell, 4-cell, 5-cell, and 8-cell divisions are depicted and the durations and synchronicities of the second and third cell cycles (s2, s3, ECC2, ECC3) and duration of blastulation (dB) are calculated using the exact timings. Snapshots were generated by the EmbryoScope and edited with GIMP

Mouse embryos (*N =* 70) were thawed and cultured in 2 consecutive repetitions of the experiment. On day 1, 1-cell stage mouse embryos (B6C3F-1 × B6D2F-1 strain) were thawed in Hepes-buffered medium (Sigma-Aldrich, UK) following the manufacturer’s protocol (Embryotech, USA). The embryos were individually placed in micro-wells of EmbryoSlides (Vitrolife, Sweden) previously prepared with 25 μl of Embryomax KSOM medium overlaid with 1.4 ml of Embryomax light mineral oil (Merck, UK) and equilibrated at 37 °C in 5% CO_2_. The embryos were cultured in the EmbryoScope time-lapse incubator (Vitrolife, Sweden) at 37 °C and 5% CO_2_ until they reached the full blastocyst stage. Each embryo was captured with an in-built camera every 15 min at 9 focal planes. Time-lapse imaging was terminated after 4 days of culture (80 h post-thaw). The EmbryoViewer software (Vitrolife, Sweden) was used to process the data. The following morphokinetic events were annotated following previously proposed guidelines [19]: pronuclei fading (tPNf); first cleavage (t2); division to the 3-cell (t3), 4-cell (t4), 5-cell (t5), and 8-cell (t8) stages; start of blastulation (tSB); and full blastocyst (tB) stages (Fig. 1). Using this information, the durations of the second (ECC2 or t4-t2) and third (ECC3 or t8-t4) cell cycles and the synchronicity of the second (s2 or t4-t3) and third (s3 or t8-t5) cell cycles, and duration of blastulation (dB) were calculated. Additionally, the ratios of the cleavage synchronicities from 2 to 4 cells (CS2–4), 2 to 8 cells (CS2–8), and 4 to 8 cells (CS4–8) were calculated as described previously [20]. Finally, the blastocysts were graded at the specific timeframe of 70.0–73.0 h (post-thaw) as good, fair, or poor morphology, based on the quality of the trophectoderm and inner cell mass as previously described [21]. Further information regarding the criteria used for the morphokinetic annotations, calculations of relative timings and synchronicities, and blastocyst grading can be found in Tables S1–S4 of Electronic Supplementary Material 1.

### Blastocyst staining

**Fig. 2:**
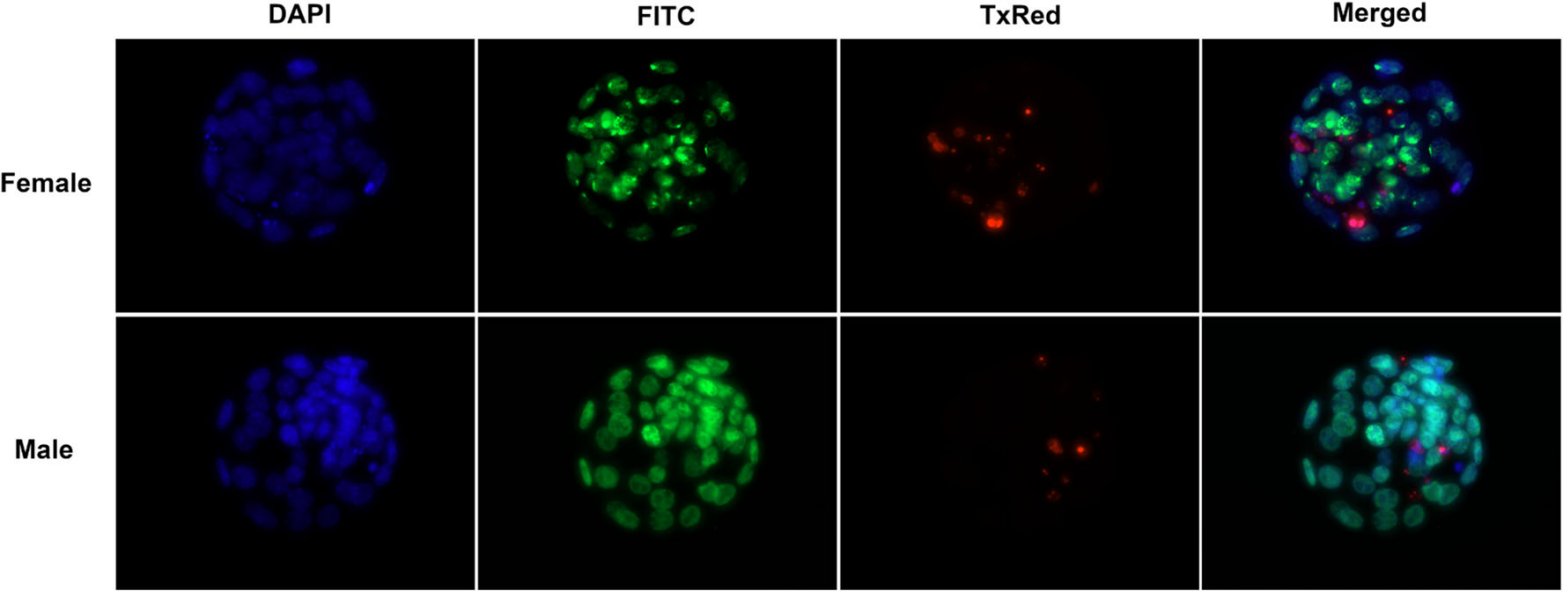
Mouse blastocysts × 40 magnification after triple staining. The DAPI nuclear dye was used to count the TCN and the TxRed dye specifically binds to broken DNA, indicating this way cell death. The apoptotic extent was calculated by dividing the apoptotic cells with the TCN (%). Gender determination was based on the molecular phenomenon of X inactivation. Histone 2a ubiquitin is enriched in the Xi in females; therefore by immuno-targeting this protein, an Xi patch appears in cells of female embryos. In the first row, a female embryo is displayed with the characteristic FITC nuclear patch whereas in the male embryo (lower row), the dye is diffused since Histone 2a ubiquitin is not concentrated in a genomic region. The figure contains 8 images generated by Volocity and edited with GIMP. The figure is intended for color on web

To exclude sex-related differences in miRNA expression as reported for other species [7, 16], sexing of embryos was carried out. Sex determination was based on the molecular phenomenon of X chromosome inactivation (XCI) which happens at the blastocyst stage in female mouse embryos. One of the earliest chromatin alterations during XCI is H2AK119 ubiquitination, meaning that the chromatin of the inactivated X chromosome (Xi) becomes enriched in repressive H2AK119ub marks [22]. Therefore, by immuno-targeting this protein, an Xi patch appears in cells of female embryos. Firstly, the blastocysts were separately placed in 4% PFA for 12 min at room temperature for fixation. After washing in 2% BSA/PBS, the Triton X-100 reagent (Sigma-Aldrich, UK) was used at a 0.1% concentration for permeabilizing the membranes. The embryos were washed and placed in drops containing the primary antibody H2AK119ub (1:200) (Cell Signaling Technology, USA) overnight under mineral oil at 4 °C. The next day, the blastocysts were incubated with the Anti-Rabbit IgG secondary antibody (1:300) conjugated with Alexa Fluor 488 (Abcam, USA) under mineral oil at room temperature for 1 h. Next, they were stained for apoptosis using the TdT-mediated dUTP-X nick end labeling method (TUNEL) with the In Situ Cell Death Detection Kit with TMR red (Merck, UK) following the manufacturer’s instructions. TUNEL staining is a well-established method for apoptosis detection in mammalian embryos, including murine [23], bovine [24], porcine [25], and human [26] embryos. Lastly, the embryos were loaded with the Vectashield Mounting Medium containing DAPI (Vector Laboratories, USA) in 10-well slides (ThermoFisher, UK) for nuclear staining. The embryos were visualized using a fluorescence microscope with the appropriate filters adjusted to correspond to the maxima excitation and emission wavelengths of the dyes (490/525 nm Alexa Fluor 488, 596/615 nm TxRed, 350/470 nm DAPI). The Volocity 3D imaging software (PerkinElmer Inc., USA) was used to capture images and annotate the total cell number (TCN) and apoptotic extent and identify the sex (Fig. 2).

### Culture media analysis

When culture was terminated, 20 μl of SCM was collected from each embryo, immediately snap-frozen in LN_2_, and stored at − 80 °C. Control samples containing culture medium not exposed to an embryo were collected from each slide. The miRNeasy Serum/Plasma Kit by Qiagen (USA) was used for miRNA extraction. The manufacturer’s instructions were followed with small alterations to the protocol aiming to increase the miRNA concentration. For this, the 20-μl samples were first diluted 1 in 2 with RNase-free water and the final elution volume was altered from 14 to 10 μl. Next, the Taqman Advanced miRNA cDNA Synthesis Kit (Applied Biosystems, UK) was used per the manufacturer’s guidelines. In the poly (A) tailing reaction, the RNase-free water was substituted with equal amount of sample to avoid dilution of the samples. The TaqMan™ Universal PCR Master Mix, no AmpErase™ UNG, and TaqMan™ Advanced miRNA Assays (Applied Biosystems, UK) were used to amplify miR-291a and miR-294 (Table 1). The samples were run in the 7500 Fast Real-Time PCR System (Applied Biosystems, UK) setting the cycling conditions as follows: 95 °C for 20 s and 40 cycles at 95 °C for 3 s followed by 30 s at 60 °C. All data were analyzed with the 7500 Software V2.3 (Applied Biosystems, UK) using the Ct values as read-outs. MiRNAs let-7b and cel-miR-39 spike-in were used for normalizing the Ct values. The Ct value of 35 was used as cutoff to capture all the potentially valid signals.

**Table 1:** Assays used for amplifying each miRNA in mouse blastocysts. The target miRNA, mirBase ID, and mature sequence are described

TaqMan miRNA probes			
	Assay name	MiRBase accession number	Mature miRNA sequence (5′-3′)
Target			
miR-291a	mmu-mir-291a-3p	MIMAT0000368	AAAGUGCUUCCACUUUGUGUGC
miR-294	mmu-mir-294-3p	MIMAT0000372	AAAGUGCUUCCCUUUUGUGUGU
Normalizers			
let-7b	hsa-let-7b-3p	MIMAT0004482	CUAUACAACCUACUGCCUUCCC
miR-39	cel-miR-39-3p	MIMAT0000010	UCACCGGGUGUAAAUCAGCUUG

### MiRNA mechanism of release

Where there was an association between apoptosis and miRNA in SCM, we decided to extend our research and find the mechanism involved. Usually, miRNA studies use miRNA mimics to cause the downregulation of in silico predicted targets in order to explain their findings. It is a common misconception to assume that miRNA levels in spent culture medium reflect on the intra-cellular content, i.e., the more miRNA you have in SCM, the more you have inside the blastocyst cells. If this was the case, using mimics is of course an acceptable approach; however, no study has proven this yet. Our next questions were therefore the following: Do embryos that release higher levels of a specific miRNA in SCM have increased levels of this miRNA inside their cells? Does the intra-cellular miRNA trigger apoptotic pathways? Or do highly apoptotic embryos actively release more miRNA extracellularly, which could then serve another function such as cell signaling?

To answer these questions, two additional experiments were carried out. Firstly, mouse embryos were cultured individually to the blastocyst stage; SCM samples and each blastocyst were collected and stored at − 80 °C (*N* = 54). SCM miRNA levels were analyzed, and based on the results, the embryos were divided into 4 groups: null, low, medium, and high miR-294. Each group contained 10 embryos from which miRNAs and total RNA were extracted using the miRNeasy Micro Kit (Qiagen, USA). MiRNAs were amplified and detected using the protocols described in the previous section. CDNA libraries were built from RNA using the High Capacity RNA-to-cDNA kit (ThermoFisher, USA) and the expression patterns of two key apoptotic factors, namely *Bcl2* and *Apaf-1* normalized with *Rpl5*, were investigated with the corresponding TaqMan™ assays using the following PCR conditions: holding at 50 °C for 2 min and 95 °C for 20 s and 40 cycles at 95 °C for 3 s followed by 30 s at 60 °C. The cutoff Ct value of 35 was used to filter out fluorescence artifacts.

In the second experiment, direct DNA damage was caused in embryos by UV radiation to explore if induced apoptosis would cause increased miRNA release. For this, mouse embryos were cultured to the early blastocyst stage (~ 70 h post-thaw) and were exposed to UV radiation by placing them on the benchtop UV Transilluminator (UVP, USA) for 5 s (wavelength 302 nm). The embryos were cultured for 16 more hours (in total 96 h post-thaw) to allow for transcriptional and translational responses to DNA damage. A control group with no UV treatment was also cultured in parallel. All blastocysts were collected and stained with TUNEL (protocol described above) to assess DNA breakage. SCM samples were collected and analyzed for miRNA expression.

### Statistical analysis

The SPSS Statistics 26 software (IBM, USA) was used for data analysis. Data distributions were checked using the Shapiro-Wilk test for normality and visual examination of the histograms. The Kruskal-Wallis test was performed to examine the distribution of apoptosis across the good, fair, and poor morphology groups. The 2^-ΔCt^ values were used for all statistical tests using the miRNA and RNA data [27]. Log transformations were carried out where necessary to achieve normal distribution of the values. Independent samples *t* tests were carried out to examine the distribution of miR-291a and miR-294 across the sex groups. Spearman’s and Pearson’s correlation coefficients were calculated for examining potential correlations between morphokinetics and apoptosis with miRNA levels in SCM. ANOVA and Tukey’s post hoc comparisons were performed to detect significant differences in miRNA and RNA expression between the null, low, medium, and high miRNA release groups in the second part of this study. A *t* test comparison was carried out to detect significance in miRNA levels between the UV-treated and control groups. A *p* value of < 0.05 was considered significant for all tests. Graphs were generated using GraphPad PRISM 8.

## Results

### MiRNA presence in SCM

From the 70 mouse embryos thawed, 59 (84%) cleaved, formed morulas, and reached the blastocyst stage by day 4 of culture. The SCM from 59 blastocysts were analyzed for the presence and levels of the miRNAs. For miR-291a, 20 samples (34%) showed amplification below the cutoff Ct value, and for miR-294, 43 samples (73%) amplified. The mean Ct for miR-291a was 30.9 ± 3.1 (STDEV) (ΔCt 25.7 ± 3.5) and for miR-294 27.8 ± 2.7 (ΔCt 22.7 ± 2.8). The remaining samples for each miRNA were either negative or amplified beyond 35 cycles thus being treated as negative samples. Blank samples, namely water and culture media not exposed to embryo culture, were also run in parallel and showed no amplification for the miRNAs.

### MiRNAs and embryo sex

**Fig. 3:**
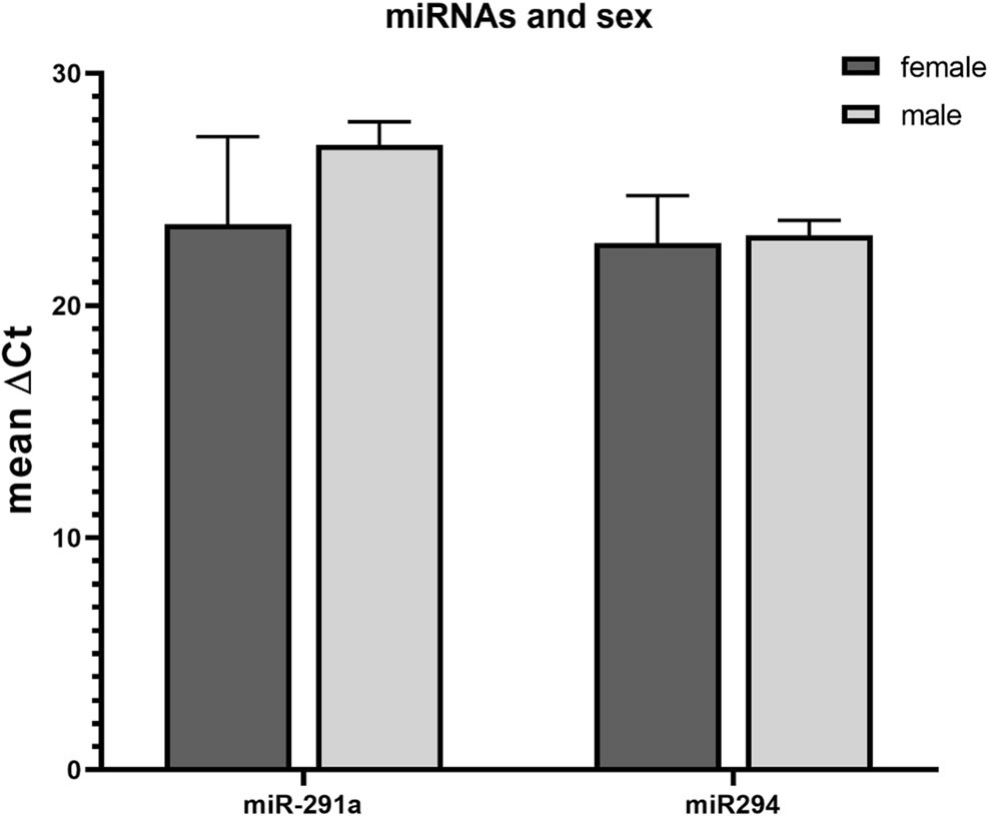
Mean ΔCt for each miRNA in male (*N =* 39) and female (*N =* 19) embryos. T-bars depict the standard deviation for each variable. The figure was created by GraphPad Prism 8

Sample size differed between sex groups with 30 male versus 19 female mouse embryos. The mean miR-291a ΔCt was 26.9 ± 3.1 for male embryos and for female embryos 23.5 ± 3.5 and miR-294 were 23 ± 3 and 22.7 ± 2 for male and female blastocysts respectively. Mean comparisons showed that miR-291a and miR-294 were released in similar levels by the embryos (*p* > 0.05) (Fig. 3). In consideration of these results, further correlation tests were not adjusted for sex-related bias.

### MiRNAs and development

Data from 59 mouse embryos were analyzed to examine the relationship between preimplantation development and miRNA levels in SCM. For this, the miRNAs were compared with absolute timings (t2, t3, t4, t5, t8, tSB, tB—normalized with tPNf) and relative timings of ECC2, ECC3, s2, s3, dB, CS2–8, CS4–8, and CS2–4. No significant correlations were found between the above developmental events and miR-291a and miR-294 levels. However, a tendency for significance was observed in the comparison of blastocyst formation timing, tB, with miR-294 (*p* = 0.051, *r* = 0.303, *N* = 42) where embryos with prolonged tB released higher levels of miR-294.

### Morphology, miRNAs, and apoptosis

**Fig. 4:**
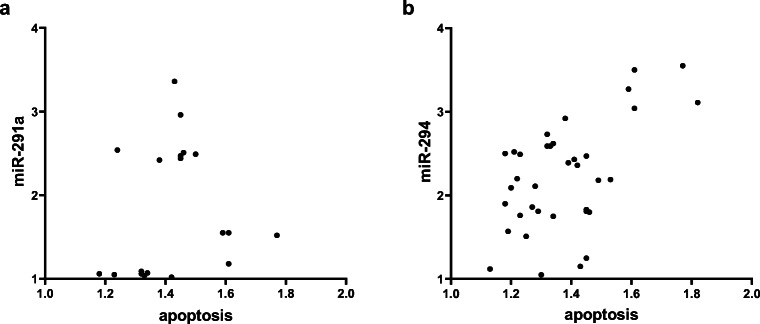
Scatter plots of miR-291a and miR-294 in relation to apoptosis. The variables are transformed using log10 (*x* + 10) to achieve Gaussian distributions. **a** There is no correlation between miR-291a and apoptosis (*N* = 21, *p* > 0.05). **b** MiR-294 and apoptosis are significantly correlated (*N* = 36, *p* < 0.001) in a positive relationship (*r* = 0.560). The figure was created by GraphPad Prism 8

After assessing the morphology of 59 blastocysts, 4 were categorized as good, 42 as fair, and 6 as poor, whereas 7 embryos had not reached the full blastocyst stage at the 70.0–73.0 interval and were not classified. Moreover, 47 blastocysts were stained for apoptosis and categorized for morphology. The average apoptotic percentage was 12% ranging from 0 up to 56%. Analysis of variance tests showed that the average extent of apoptosis is similar across the three morphology groups (good = 21 ± 11%, *N* = 4; fair = 15 ± 7%, *N* = 37; poor = 9 ± 4%, *N* = 6) (*p* > 0.05). The SCM levels of miR-291a were not associated with apoptosis (*p* > 0.05) (Fig. 4a). However, miR-294 was significantly correlated with the extent of apoptosis (*N* = 36, *p* < 0.001) with Pearson’s correlation revealing a positive relationship between these variables (*r* = 0.560) (Fig. 4b).

### MiR-294 mechanism of release

**Fig. 5:**
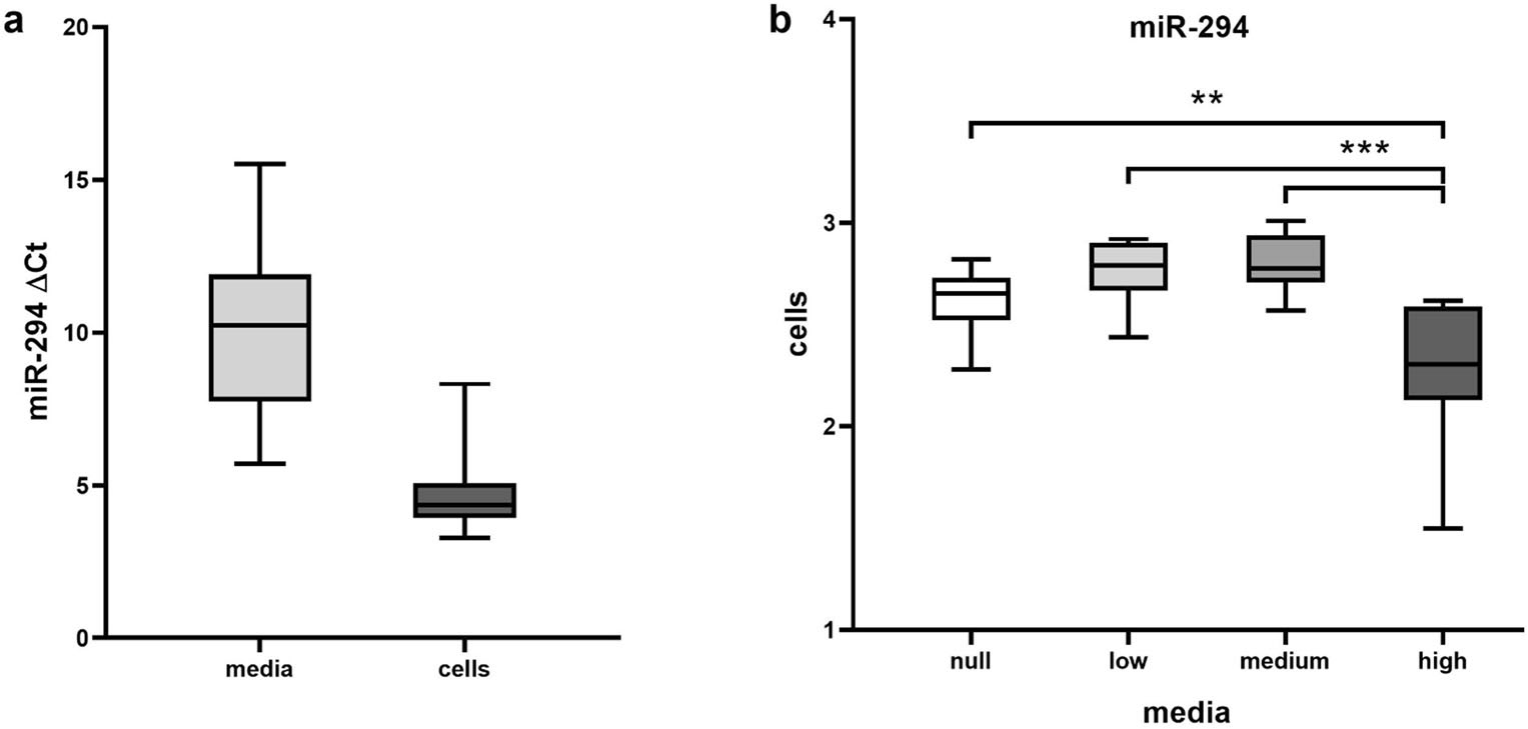
**a** MiR-294 average ΔCt in spent culture media samples (*N* = 30) and in blastocyst cells (*N* = 40). **b** Comparison of miR-294 levels (log10 (*x* + 10) values) between null, low, medium, and high miR-294/SCM groups. The boxplots contain the middle 50% of the values (Q3–Q1) and the median (horizontal line) and the whiskers depict the maximum and minimum values for each group. Significant differences in expression between the high and the null, low, and medium groups are marked with asterisks. The figure was created by GraphPad Prism 8

**Fig. 6:**
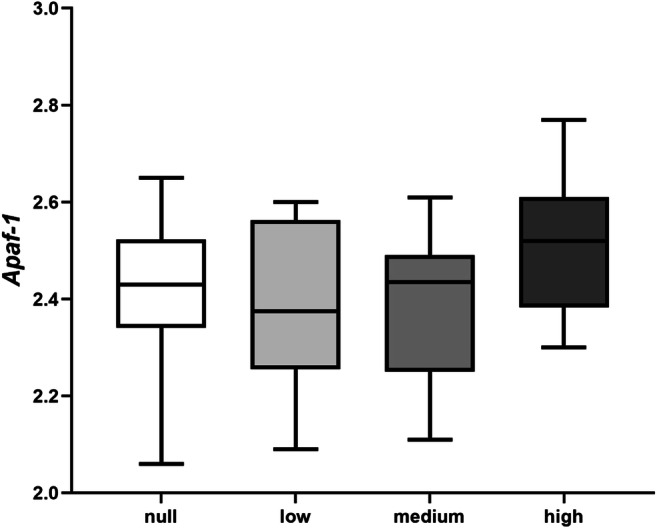
Comparison of intra-cellular *Apaf-1* expression (log10 (*x*) values) between the null, low, medium, and high miR-294 release groups. The boxplots contain the middle 50% of the values (Q3–Q1) and the median (horizontal line) and the whiskers depict the maximum and minimum values for each group. Embryos from the 4 groups expressed similar levels of *Apaf-1* (*p* > 0.05). The figure was created by GraphPad Prism 8

From the 54 SCM samples, 13 were null for miR-294 (76% amplification) and the average Ct from the rest of the samples was 27.9 ± 2.2 (ΔCt 8.9 ± 2.1). By calculating the Q1 and Q3 quartiles of the population, the blastocyst samples were categorized in null (Ct > 35, zero expression), low (ΔCt 11.6 ± 0.8), medium (ΔCt 9.1 ± 0.6), and high (ΔCt 6 ± 0.9) miR-294 groups (*N* = 10 for each group). After miRNA extraction, miR-294 levels were compared with ANOVA which detected significant difference in expression between the groups (*p* < 0.001). Post hoc comparisons showed that it was the high group that released significantly more miR-294 compared with the null (*p* < 0.01), low (*p* < 0.001), and medium (*p* < 0.001) groups (Fig. 5). Furthermore, *Bcl2* expression was null for all 40 blastocyst samples. However, *Apaf-1* was expressed in embryonic cells with an average Ct of 33.4 ± 0.6 (ΔCt 5.3 ± 0.6) in the null group, 33.7 ± 0.7 (ΔCt 5.4 ± 0.6) in the low group, 32.7 ± 0.7 (ΔCt 5.4 ± 0.5) in the medium group, and 33.4 ± 0.7 (ΔCt 4.9 ± 0.4) in the high group. ANOVA testing showed that the expression of *Apaf-1* was similar between the groups (*p* > 0.05) (Fig. 6).

**Fig. 7:**
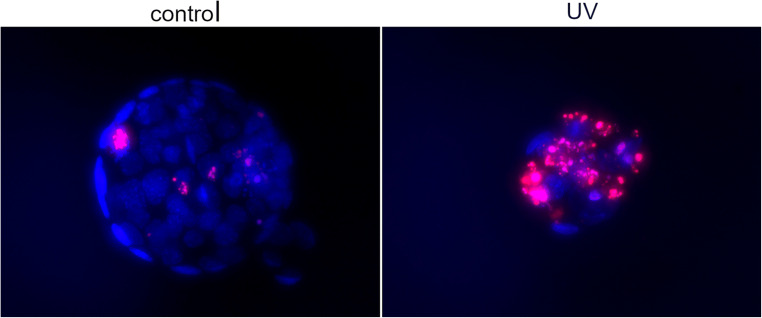
Induced DNA breakage using UV radiation. On the left image, a control blastocyst with few apoptotic cells, and on the right image, a UV-treated embryo with reduced TCN and many apoptotic cells. Embryos from both groups were cultured in parallel and for the same period. Contains two images generated by Volocity and edited with GIMP. The figure is intended for color on web

**Fig. 8:**
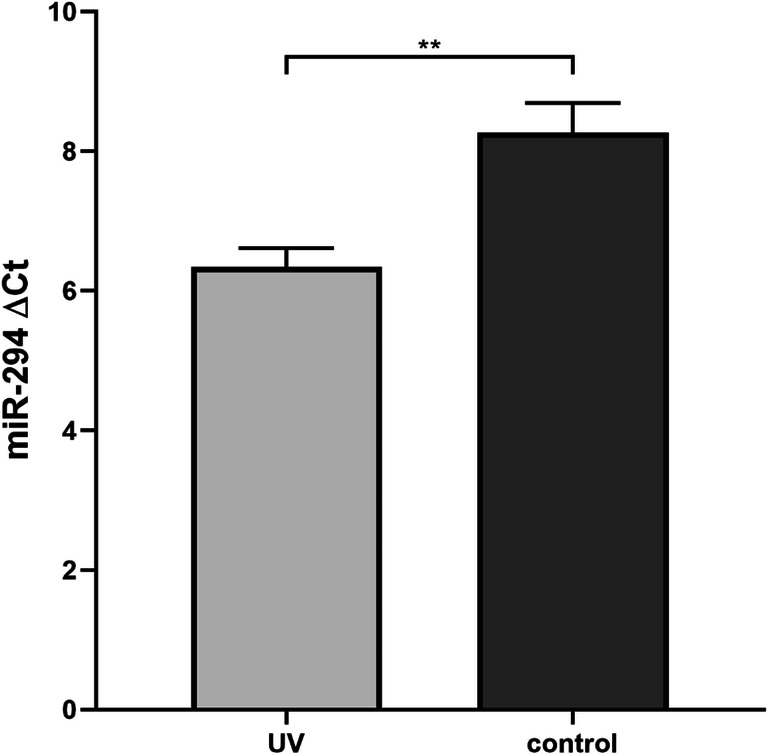
Average miR-294 ΔCt between the UV-treated and control embryos. The embryos treated with 5 s of UV (*N* = 15) released significantly more miR-294 in the SCM compared with the control group (*N* = 13) (*p* < 0.01). T-bars show the standard deviation of the values. The figure was created by GraphPad Prism 8

For the UV experiment, a total of 15 blastocysts were subjected to UV radiation (302 nm) for 5 s and 13 blastocysts were used as control of the study. Using TUNEL staining, the direct effects of UV on DNA damage was verified, with the UV group showing higher extent of apoptosis (and reduced cell proliferation) compared with the control group (Fig. 7). The average miR-294 Ct in the SCM of the UV group was 25.1 ± 1.1 (ΔCt 6.3 ± 1) and in the control group 27.1 ± 1.5 (ΔCt 8.3 ± 1.5). The results from the *t* test showed that the blastocysts with UV-induced DNA damage released significantly more miR-294 in the media (*p* < 0.01) (Fig. 8).

## Discussion

To the best of our knowledge, this is the first report investigating specific miRNAs in culture media from singly cultured embryos in association with morphokinetics and apoptosis. The main finding of this study is that miR-294 is directly correlated with the extent of apoptosis in mouse embryos. Our results overall suggest that increased miR-294 is a result of extended apoptosis, which implies that apoptosis-triggered mechanisms regulate the transcription, packaging, and transport of miR-294 outside the embryonic cells.

### MiR-291a and miR-294 are released in SCM of mouse blastocysts

In our study, we used the mouse model and examined the extracellular release of two miRNAs, namely miR-291a and miR-294, as candidate markers of embryonic development and quality. These miRNAs were specifically selected because they are expressed in embryonic cells [28] and more importantly they are amongst the earliest miRNAs expressed de novo during mouse embryonic development [3]. MiR-290 members have numerous gene targets that regulate key processes of development such as cell cycle, differentiation, and programmed cell death [29]. For instance, both miR-291a and miR-294 target *Cdkn1a* which is a regulator G1/S transition and cellular proliferation (www.targetscan.org) and they also regulate apoptotic mediators *Casp2* and *Ei24* whose actions promote cellular death, autophagy, and degradation [30]. Regarding their release, although the miR-290 cluster is mouse-specific, there are reports about its homologue cluster miR-371/373 in humans [31]. Specifically, two studies reported the release of miR-371 by human blastocysts [7, 17].

As expected, both miR-291a and miR-294 were present in detectable levels in the culture media of mouse embryos in our study. These findings agree with previous publications showing that mammalian blastocyst-stage embryos release microRNAs in their environment, which is known for bovine [10, 16] and human [13, 17, 18] embryos. To the best of our knowledge, there are no publications showing similar findings for the mouse embryo yet. Importantly, in contrast to other studies that pool SCM samples using different grouping criteria [11, 13, 16, 17], we were able to isolate and quantify miRNAs from singly cultured embryos. This is preliminary evidence for future studies which would likely explore the value of miRNA SCM analysis for noninvasive embryo assessment.

### Extended apoptosis causes the release of miR-294 in SCM

Since the miR-290-295 family members are expressed in mouse embryos and have roles in DNA repair mechanisms [32], it was hypothesized that the release of miR-291a and miR-294 in culture media would reflect on the apoptotic status of the embryos. Indeed, it was found that miR-294 is positively correlated with apoptosis in mouse blastocysts. To better understand this association, we investigated the mechanism involved.

The first possibility was that the actions of these miRNAs on apoptosis regulation led to the observed difference, meaning that high intra-cellular content of miR-294 altered the actions of factors which promoted DNA damage and apoptosis. For instance, target prediction analysis shows that miR-294 is involved in the post-transcriptional regulation of the apoptosis inhibitor *Bcl2* (www.targetscan.org)—a regulatory mechanism which could possibly explain our finding. In this scenario, the embryos which release more miR-294 have more miR-294 in their cells which suppresses *Bcl2* expression and overall promotes apoptosis. To investigate this, firstly, we compared the intra- and extracellular miR-294 levels in 40 blastocysts. Surprisingly we found that embryos releasing more miR-294 in SCM had significantly less miR-294 in their cells compared with the null, lower, and medium release groups. Additionally, the expression of *Bcl2* was analyzed in the same samples; however, it was undetectable. Because of this, the apoptotic regulator *Apaf-1* which is involved in the same pathway as *Bcl2* was chosen as an alternative apoptotic marker. *Apaf-1* acts as a key activator at the last regulatory level of the *Bcl2* apoptotic pathway through direct activation of caspases which then degrade cellular components. *Apaf-*1 was expressed in detectable levels in all the blastocysts; however, its expression was similar across the groups. Considering the above, no further conclusions could be made regarding the specific apoptotic pathway. Overall, these results clearly suggest that miRNA levels in SCM, at least for the specific miRNA, are not positively correlated with cellular content. Possibly, the reason why embryos which release more miR-294 have less of this miRNA in their cells is due to intense miRNA packaging and exosome release in the surrounding micro-environment. It is therefore likely that molecular pathways trigger miRNA release from blastocysts and not the other way around. In the light of the above, the use of miRNA mimics in this study was rejected and the mechanism of miR-294 release was further investigated.

The second possibility was that increased apoptosis triggered the release of miR-294. To investigate this, UV radiation was used to cause DNA damage in blastocysts. A similar methodology has been reported previously for UV-induced apoptosis in sea urchin embryos [33]. Nevertheless, our study describes original work using UV radiation to “mimic” the final stages of apoptosis in order to study apoptosis-triggered mechanisms in mammalian embryos. With this experiment, we aimed to investigate if it is the process of apoptosis that enhances the packaging and release of miR-294 from the embryos. The embryos were subjected to UV radiation specifically when they reached the early blastocyst stage for two reasons. Firstly, it was important not to compromise embryo survival and blastocyst formation because it is mainly the trophectoderm population that actively releases miRNAs in the extracellular environment [18]. Additionally, by UV-treating the embryos at the final stages of development, DNA repair mechanisms which are activated in the cells had minimum time to reverse the effect [34]. On the other hand, it was crucial for this study to maintain embryo viability for enough time to allow for transcriptional responses to DNA damage and, more specifically, cause enhanced transcription, packaging, and release of miR-294. In parallel, it was important to cause high damage in order to observe the maximum effect of the treatment. After some optimization steps, it was found that UV radiation for 5 s at 302 nm caused direct DNA breakage in the blastocysts without instantly killing them. The increase in DNA breaks and cell apoptosis was verified using TUNEL staining and miR-294 in SCM was measured. In agreement with the hypothesis, the UV-treated embryos released significantly more miR-294 in the media compared with the control group. Overall, these results show that extended apoptosis triggers miR-294 release, providing thus mechanistic evidence that apoptosis is the causal factor of intense miR-294 release from mouse blastocysts.

It is unlikely that increased miRNA levels in SCM are a result of membrane fragmentation/lysis but rather high miR-294 is a result of intense active release from embryonic cells. In our experiments, we monitored and recorded cell lysis using time-lapse and no association was found with miRNA levels (data not shown). Moreover, increasing evidence shows that extracellular release of miRNAs is a regulated process, rather than a result of cell injury, and these molecules have hormone-like properties in the sense that they cause autocrine, paracrine, and/or endocrine responses [35]. Indeed, embryos secrete miRNAs which are internalized by endometrial cells and regulate the transcription of genes that either promote or inhibit implantation [16, 17]. Bi-directional communication is also possible via miRNAs as shown for endometrium-secreted miR-30d and human embryos [36]. Considering the findings of this study and the available literature, we suggest that increased apoptosis triggers the active release of miR-294 which possibly acts as a signaling molecule for communication with the endometrium. For instance, it could be that miR-294 inhibits the attachment of the embryo by downregulating cell adhesion factors, such as *Pcdh* and *Cdh* cadherin genes, and promoting cell cycle arrest and apoptosis through *E2f2* and *Tnf* regulation (www.targetscan.org). Possibly, poor-quality embryos secrete miR-294 to inhibit implantation and overall cause rejection of the embryo “saving” this way the maternal energy investment in establishing pregnancy. Although this assumption needs to be further investigated, it has been shown previously that embryo-secreted miRNAs affect attachment and adhesion to the endometrium [17] which further strengthens our suggestion that miR-294 could act as a signaling molecule for endometrial attachment.

In addition to the aims of this study, we provide preliminary evidence on the use of miRNAs as noninvasive markers of embryos’ apoptotic status. In embryology, determining the extent of apoptosis is very important considering that high apoptosis has negative effects on embryo survival and reflects on implantation and pregnancy rates [37]. However, it is known that apoptosis cannot always be assessed morphologically, and to further support this, we carried out blastocyst scoring and compared it with the number of apoptotic cells in each embryo (no correlation found). Additionally, other quality markers such as multinucleation at the t3-t2 and t5-t4 intervals, fragmentation at the 4- and 8-cell stages, and blastomere symmetry at the 2-, 4-, and 8-cell stages were annotated (data not shown), but again no association was found with apoptosis. Moreover, although recent evidence links the levels of another miRNA, miR-30c, with apoptosis in bovine embryos [12], our study is the first report where SCM samples are individually processed and analyzed, with miRNA levels directly correlated with apoptosis in the same embryos. Therefore, the presented study could be a reference for future research where extracellular miRNAs are used for embryo (de-)selection purposes.

## Conclusions

In summary, miRNAs are present in SCM and are detectable in minimum volume of individually cultured embryo media. MiR-294 in spent media is directly linked to the apoptotic percentage in mouse blastocysts. Notably, it is increased apoptosis that triggers the mechanism of miR-294 release in the surrounding micro-environment. Possibly this is a secretory mechanism which is activated for the inter-cellular communication with the endometrium and could play a role in embryo attachment. Further experiments in which high miR-294 release embryos are transferred to the endometrium would provide a clear understanding of the role of miR-294 in implantation. Overall, miRNAs are important regulators of cell processes and are candidate molecules for future reproduction research.

## Electronic supplementary material

ESM 1 (DOCX 17 kb)
