# Supplementary material for: Apoptosis triggers the release of microRNA miR-294 in spent culture media of blastocysts
Source: J Assist Reprod Genet. 2020 May 21;37(7):1685–94. doi: 10.1007/s10815-020-01796-5 (PMC7376808; doi:10.1007/s10815-020-01796-5)
Supplement: Supplementary file 1 — (DOCX 17 kb) [file 10815_2020_1796_MOESM1_ESM.docx]

| **Annotations** | **Cell stage** | **Criteria** |
| --- | --- | --- |
| tPNf | Pronuclear fading | First frame in which both pronuclei disappear |
| t2, t3, t4, t5, t8 | 2-cell, 3-cell, 4-cell, 5-cell, 8-cell | First frame in which the 2, 3, 4, 5, or 8 cells are clearly separated by membranes accordingly |
| tSB | Small blastocoel | First sign of blastocoel formation |
| tB | Blastocyst | Last frame before the embryo starts pushing against the ZP |

**ELECTRONIC SUPPLEMENTARY MATERIAL 1**

**Table S1.** Exact timings, developmental stage, and criteria used to annotate the time-lapse parameters of the embryos.

| **Relative timings** | **Morphodynamic event** | **Calculation** |
| --- | --- | --- |
| ECC2 | Duration of second cell cycle | t4-t2 |
| ECC3 | Duration of third cell cycle | t8-t4 |
| s2 | Synchronization of cell divisions | t4-t3 |
| s3 | Synchronization of cleavage pattern | t8-t5 |
| CS2-4 | 2- to 4- cell stage cleavage synchronicity | (t4-t3) / (t4-t2) |
| CS2-8 | 2- to 8- cell stage cleavage synchronicity | ((t3-t2) + (t5-t4)) / (t8-t2) |
| CS4-8 | 4- to 8- cell stage cleavage synchronicity | (t8-t5) / (t8-t4) |
| dB | Duration of blastulation | tB-tSB |

**Table S2.** Calculations of developmental events using the exact timings described above.

| SART grade | Embryo grade (ICM/TE) |
| --- | --- |
| Good | AA, AB |
| Fair | BA, BB, BC |
| Poor | CB, CC |

**Table S3.** Simplified SART system used to categorize the blastocysts in three morphology groups.

|  | ICM | TE |
| --- | --- | --- |
| A | Prominent, easily discernible, with many cells that are compacted and tightly adhered together | Many cells forming a cohesive epithelium |
| B | Easily discernible, with many cells that are loosely grouped together | Few cells forming a loose epithelium |
| C | Difficult to discern, with few cells | Very few cells |

**Table S4.** ICM and TE characterization criteria used to score the blastocysts.
